# Association between Healthy Eating Index-2015 scores and probable sarcopenia in community-dwelling Iranian older adults: a cross-sectional study

**DOI:** 10.1017/jns.2021.12

**Published:** 2021-03-25

**Authors:** Zahra Esmaeily, Zahra Tajary, Sharzad Daei, Mahshid Rezaei, Atefeh Eyvazkhani, Ahmad Reza Dorosty Motlagh, Andriko Palmowski

**Affiliations:** 1 Department of Community Nutrition, School of Nutritional Sciences and Dietetics, Tehran University of Medical Sciences, Tehran, Iran; 2 Department of Nutrition, Science and Research Branch, Islamic Azad University, Tehran, Iran; 3 Department of Rheumatology and Clinical Immunology, Charité – University Medicine Berlin, Berlin, Germany

**Keywords:** Aging, HEI-2015, Iranian, Probable sarcopenia, Sarcopenic

## Abstract

Sarcopenia is associated with frailty and disability in older adults. Adherence to current dietary guidelines in addition to physical activity could prevent muscle wasting and weakness. The Healthy Eating Index-2015 (HEI) is a tool to assess diet quality. We aimed to investigate the association between HEI scores and probable sarcopenia (PS) among older adults in Tehran. 201 randomly selected older adults were included in this cross-sectional study between May and October 2019 in Tehran, Iran. A previously validated semi-quantitative food frequency questionnaire was used to estimate HEI scores and dietary intake. Handgrip strength (HGS) was measured to evaluate the PS. Statistical evaluation included descriptive analysis, logistic and linear regression. Those probably suffering from sarcopenia had significantly lower HEI scores (*P*=0⋅02). After adjusting for confounders, HEI scores and HGS were still significantly associated (adjusted *R*^2^=0⋅56, slope *β*=0⋅03, *P*=0⋅09). Older adults with a low PS had a higher ratio of monounsaturated and polyunsaturated to saturated fatty acids (*P=* 0⋅06) and ingested less added sugars and saturated fats (*P*=0⋅01 and *P*=0⋅02, respectively). Furthermore, consuming more total protein foods correlated positively with muscle strength (*P*=0⋅01, *R*=0⋅18). To sum up, HEI scores were associated with PS, measured by HGS, indicating that adhering to the HEI might improve muscle strength in aging individuals.

As life expectancy is increasing, there is a need for studies on health problems of the aging population^(1)^. Aging goes hand in hand with physical changes which may lead to both decreased muscle mass and decreased physical function. In pathologic form, this is called sarcopenia^(2)^. According to the European Working Group of Sarcopenia in Older People (EWGSOP), sarcopenia can be defined as a decrease of muscle mass and physical activity or a low muscle mass with decreased muscle strength in older adults. Probable sarcopenia (PS) can be estimated by evaluating muscle strength^(3)^. Decreasing muscle strength starts to occur gradually in the thirties and further accelerates by the age of fifty^(4)^. Sarcopenia has different causes, which include some diseases, decreased calorie intake, lack of physical activity, low muscle perfusion, mitochondrial dysfunction, decreased anabolic hormones and increased pro-inflammatory cytokines^(5)^. It can lead to major health problems in older adults, e.g., muscle weakness increases the odds of falling^(6)^.

Several studies have been published concerning the role of nutrient intake and muscle weakness or sarcopenia. However, assessing a nutrient by itself may give only a little information about diet quality^(7,8)^. Few other studies have focused on dietary patterns rather than on a particular nutrient to examine relationships between diet and health status^(9–11)^.

Assessing dietary pattern quality is one way to demonstrate a person's dietary status. The Healthy Eating Index (HEI) was developed by the United States Department of Agriculture (USDA) and is based on Dietary Guidelines for Americans (DGAs)^(12)^. Higher HEI scores indicate better adherence to the DGAs^(13)^.

Previous studies have evaluated associations between HEI scores and the risk of several morbidities. The prevalence of metabolic syndrome decreased and bone mineral density increased in Iranian women with high HEI scores. In a case-control study by Varkaneh *et al.*, high HEI scores were associated with a decreased risk of breast cancer^(14–16)^. Also, several dietary patterns have already been studied in the field of sarcopenia and muscle weakness. Studies showed that a Mediterranean diet is significantly associated with low odds of sarcopenia progression in contrast to pro-inflammatory diets^(10,14)^. Other studies suggested that adherence to the HEI and Mediterranean diets might play a role in preventing muscle weakness in the elderly^(17,18)^. Given that the scarce number of Iranian studies on the association of diet with sarcopenia and abnormal handgrips strength, and the necessity of studies about improving lifestyle factors of older adults, the present study aimed to investigate the association of HEI values with PS in community-dwelling Iranian older adults in the Tehran district of Iran.

## Methods

### Data collection

This cross-sectional study was conducted between May and October 2019 in randomly selected older adults (≥60) residents of Tehran, Iran. Eligible participants were people with no changes in their dietary habits during last year, who could walk without any helping equipment, with no artificial or prosthetic limbs and with no history of congestive heart failure, chronic obstructive pulmonary disease, chronic renal failure, cirrhosis or active cancer.

A pilot study was conducted in 100 older adults to check the reliability of questionnaires and also to calculate the sample size, which was calculated as follows with a type I error (*α)*=0⋅05 and type II error *(β*)=80 %:
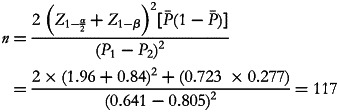


To include 117 participants with a high PS, 191 overall participants were originally needed. We sought to include 235 older adults to further increase statistical power. After asking about their dietary habits, physical and medical situations, 34 of the 235 individuals did not meet the inclusion criteria; therefore, 201 subjects eventually took part in the present study.

For sampling, Tehran was divided into five regions: East, West, North, South and city centre. Sampling was done in two stages. Firstly, a list of health centres – which participants attend for routine check-ups – was collected from every region and centres were selected by cluster sampling. Secondly, sampling was started in the first centre and continued until we found enough older adults with a high PS. We took written consent from the participants and gathered information on demographics, physical activities and socioeconomic and medical status. The amount of physical activity was estimated by asking participants about the daily average time used to exercise, jog or do other sports. Socioeconomic status was ascertained by gathering data about education and participants’ economic status. For determining the economic status, we used a 9-item questionnaire (possession of house, car, side-by-side refrigerator, washing machine, dishwasher, laptop/personal computer, sofa, microwave and handmade carpet)^(19)^ and asked about house and car ownership as we suspected to encounter refusals to declare monthly income. The participants’ economic status was categorised as Very bad: ≤3 items without any personal home and car. Bad: ≤3 items with personal home or car. 4–6 items without personal home and car. Average: 4–6 items with personal home or car. 7 items ≤ without a personal home and car. Good: 7 ≤ items with personal home or car. Very good: 7 items ≤ with personal home and car.

The present study was approved by the ethics committee of Tehran University of Medical Sciences. The protocol number of the local ethics committee was IR.TUMS.VCR.REC.1398.476.

### Anthropometric measurements

Measurements included waist, hip and mid-upper arm circumferences (MUAC), height and weight. Considering these measurements, body mass index (BMI), waist-to-hip ratio (WHR) and waist-to-height ratio (WHtR) were calculated. Subjects were considered underweight and overweight/obese if their BMI values were lower than 23⋅5 and higher than 30⋅9 kg/m^2^, respectively^(20)^. Abdominal obesity was defined as waist circumference higher than 102 and 88 cm in men and women, respectively. Besides, women with a WHR higher than 0⋅85 and subjects with WHtR higher than 0⋅6 were considered abdominally obese^(21,22)^. Of note, WHR was not used for men because of cultural and religious issues. Weight (kg) and height (cm) were measured with light clothes and no shoes on with a Camry EB9011 scale (Camry Co) and a Fibre-Glass tape measure, respectively. Waist circumference was the midway between the lowest rib and the iliac crest; hip circumference was the maximum circumference of the hip when a participant stood firmly. The circumference of the mid-upper arm was measured on the non-dominant side with the elbow flexed by 90° midway between scapula and olecranon. Circumferences were measured by a Fibre-Glass tape measure as well.

### Dietary data collection

In the present study, we used a previously validated semi-quantitative food frequency questionnaire (FFQ) with 147 items^(23)^ for assessing dietary intake during the last year. Main items included intake of bread and grains, legumes, meat and meat-derived products, poultry, fish, eggs, dairies, kinds of butter, vegetables, pickles, fruits and fruit juices, oils, seeds and nuts, added sugar, drinks, spices and salt. Both frequencies and quantities of every item were asked. Questionnaires were completed by trained dietitians.

### Calculation of HEI-2015 scores

A simple HEI scoring algorithm method was used in the present study. In this method, thirteen components containing total fruits, whole fruit, total vegetables, greens and beans, whole grains, dairy, total protein foods, seafood/plant proteins, fatty acids, refined grains, sodium, added sugars and saturated fats (including two categories for adequacy and moderation) receive scores. Every component has a minimum score of 0 and a maximum score of 5 or 10; amounts for gaining these scores have been reported elsewhere^(12)^. After scoring each component, the scores were summed up to obtain every individual's final score (ranging between 0 and 100). Reported amounts were converted to cups or ounces according to the scoring system. For converting to ounce, we divided it by 28⋅35; for converting to cup, we used the Food Patterns Equivalent Database (FPED)^(24)^.

### Probable sarcopenia

According to the EWGSOP2, the presence of PS was surrogately measured by handgrip strength (HGS)^(3)^. HGS was quantified by a squeeze dynamometer (Saehan SH5008, Co, Seoul, Korea). Participants sat on a chair with the arm flexed at 90° and pressed the dynamometer three times with each hand with maximum power and held it for 10 s. 30 s of rest were allowed between every attempt. Finally, the average maximum strength of each hand was defined to be the participant's HGS. The above-mentioned dynamometer has not been used in previous studies; thus, for assessing its accuracy, it was tested against a Jamar dynamometer, which is the gold standard also included in the EWGSOP2 standards^(3)^. Results of this comparison indicated that by multiplication by 1⋅6, we yielded the value estimated by the Jamar dynamometer. Consequently, participants were defined to have a high probability of PS if their HGS was <16⋅5 kg (men) and <10 kg (women).

### Statistical analysis

IBM SPSS software, version 16, was used for statistical analysis. The Kolmogorov–Smirnov test was performed to assess if variables were normally distributed. Mean ± standard deviation (sd) and frequencies (%) are reported for continuous and nominal variables, respectively. HEI-2015 was divided into quartiles for assessing dietary quality. Student's *t* test and analysis of covariance (ANCOVA) were used to test for statistically significant differences in continuous variables between probably sarcopenic and non-sarcopenic participants; the χ^2^ test for assessing differences of participants in the HEI quartiles. Binary logistic regression was performed for evaluating the association between HEI and PS and odds ratio (OR) calculation. Finally, a multiple linear regression model was used to adjust for confounders of HGS to assess the actual relationship between HEI and HGS. The level of statistical significance *α* was set at 0⋅1 for the multiple linear regression and 0⋅05 for other tests.

## Result

### Participant characteristics

We finally included 201 participants. In Table 1, we summarised the main characteristics of these participants. They were on average 66 years old, had a daily physical activity of 32 min, and a BMI of 29 kg/m^2^. 46 (23 %) participants were men, 155 (77 %) were women. Cardiovascular diseases and skeletal disorders were the most prevalent diseases. 123 (61 %) subjects were considered to be probably sarcopenic judging from HGS. Participants with a high PS were significantly older (67⋅54 *v.* 63⋅9 years, *P* < 0⋅0001) and had a significantly worse economic status compared with subjects with normal HGS (*P*=0⋅002). Furthermore, subjects with a history of cardiovascular disorders and those taking cardiovascular medication tended to be more often probably sarcopenic than those without (*P*=0⋅004 and *P* < 0⋅0001, respectively).

**Table 1.** Participant characteristic

Variables	Non-sarcopenic (*N*=78)		PS (*N*=123)		*P*-value*
	Mean	sd	Mean	sd	
Age (year)	63·9	3·66	67·54	5·94	<0·0001
Postmenopausal age (year)	47·35	4·86	47·91	5·64	0·53
Physical activity (min)	37·20	42·3	29·64	42·3	0·07
Weight (kg)	74·16	9·96	72·2	11·56	0·22
Height (m)	1·59	0·08	1·58	0·09	0·17
Waist circumference (cm)	97·67	8·92	97·28	10·38	0·79
MUAC (cm)	32·5	2·09	31·56	3·01	0·02
BMI (kg/m^2^)	29·26	4·07	28·82	4·09	0·45
WHtR	0·61	0·07	0·61	0·08	0·7
WHR^a^	0·88	0·06	0·86	0·1	0·25
HGS (kg)	13·29	3·52	9·16	3·03	<0·0001
	*N* (%)		*N* (%)		
Sex					0·12
Male	14 (30·4)		32 (69·6)		
Female	64 (41·3)		91 (58·7)		
Marital status					0·16
Married	61 (41·8)		85 (58·2)		
Other	17 (27·33)		38 (72·67)		
Head of the family					0·18
Father	15 (32·6)		31 (67·4)		
Mother	62 (41·9)		86 (58·1)		
Education					0·14
High school or lower	52 (36·1)		92 (63·9)		
University	26 (45·6)		31 (54·4)		
Economical statues					0·002
Very bad	7 (21·2)		26 (78·8)		
Bad	20 (48·8)		21 (51·2)		
Average	9 (25)		27 (75)		
Good	9 (26·5)		25 (73·5)		
Very good	32 (57·1)		24 (42·9)		
Supplements					
Vitamin D	56 (40·9)		81 (59·1)		0·24
Multivitamins	32 (41)		46 (59)		0·36
Minerals	39 (39)		61 (61)		0·52
Disorders					
Diabetes	19 (38)		31 (62)		0·52
Cardiovascular	33 (29·5)		79 (70·5)		0·004
Pulmonary	7 (35)		13 (65)		0·46
Renal	4 (21·1)		15 (78·9)		0·07
Skeletal	46 (34·8)		86 (65·2)		0·08
Psychological	19 (31·7)		41 (68·3)		0·12
Medication					
Diabetes	14 (33·3)		28 (66·7)		0·28
Cardiovascular	25 (26)		71 (74)		<0·0001
Skeletal	17 (32·7)		35 (67·3)		0·2
Psychological	11 (42·3)		15 (57·7)		0·41
BMI status					0·49
Underweight	4 (36·4)		7 (63·6)		
Normal	50 (38·8)		79 (61·2)		
Overweight	24 (39·3)		37 (60·7)		
WC status			0·11		
Normal	17 (30·9)		38 (69·1)		
Abdominal obesity	61 (41·8)		85 (58·2)		
WHtR statues					0·19
Normal	31 (36·9)		53 (63·1)		
Abdominal obesity	47 (40·2)		70 (59·8)		
WHR statues^a^					0·38
Normal	23 (39)		36 (61)		
Abdominal obesity	41 (42·7)		55 (57·3)		

HGS, handgrip strength; sd, standard deviation; MUAC, mid-upper arm circumference; BMI, body mass index; WC, waist circumference; WHtR, waist to hight ratio; WHR, waist to hip ratio.

a Calculated in women.

* *P* < 0.05; Student's *t* test was used for comparing mean differences of quantitative variables, and χ^2^ test was used for qualitative variables.

### HEI-2015 and handgrip strength

Mean HEI scores differed significantly between normal [60⋅55 (±9⋅85)] and probably sarcopenic [56⋅88 (±11⋅48)] groups by 3⋅67 (*P*=0⋅02). Associations between HEI components and PS are shown in Table 2. The probable-sarcopenic subjects had significantly lower scores of added sugars and saturated fatty acids. The assessment of the correlation between components of the HEI and HGS showed that there is a positive correlation between the scores of total protein foods (*R*=0⋅18, *P*=0⋅01), fatty acids (*R*=0⋅17, *P*=0⋅02), added sugars (*R*=0⋅13, *P*=0⋅06) and saturated fats (*R*=0⋅17, *P*=0⋅02) – participants with higher intake of total protein foods, poly- and monounsaturated fatty acids (PUFAs and MUFAs), and low intake of added sugars and saturated fats had significantly higher HGS. There was no statistically significant association between HEI quartiles and PS (Table 3). However, we found a reduction in proportions of probable-sarcopenic participants from 72⋅5 % in the lowest quartile to 54 % in the highest quartile. Those in the top HEI quartile were 59 and 69 % less likely to have PS in crude and adjusted models, respectively, in comparison to those in the lowest quartile (Table 4).

**Table 2.** HEI-2015 and the component scores across probable and non-sarcopenic subjects

Variables (mean ± sd)	HGS				Maximum scores of every item	*P*-value*	*P*-value**
	PS (*N*=123)		Non-sarcopenic (*N*=78)				
	Mean	sd	Mean	sd			
HEI-2015	56·88	11·48	60·55	9·85	100	0·02	0·003
Total fruits^a^	4·79	0·69	4·83	0·69	5	0·33	0·99
Whole fruits^b^	4·95	0·29	4·92	0·49	5	0·29	0·52
Total vegetables^c^	4·7	0·77	4·82	0·58	5	0·13	0·23
Greens and beans^d^	4·87	0·57	4·88	0·49	5	0·47	0·81
Whole grains	0·2	0·25	0·26	0·33	10	0·09	0·11
Dairy	6·8	2·9	6·93	2·83	10	0·38	0·82
Total protein foods^e^	4·11	1·12	4·29	0·95	5	0·12	0·33
Seafood/plant proteins^f^	2·46	1·4	2·56	1·37	5	0·32	0·86
Fatty acids^g^	4·1	3·48	5·02	3·12	10	0·06	0·03
Refined grains	1·04	2·48	1·25	2·85	10	0·29	0·14
Sodium	5·75	3·81	5·69	3·76	10	0·46	0·82
Added sugars	6·15	3·82	7·44	3·37	10	0·01	0·03
Saturated fats	6·79	3·64	7·89	2·79	10	0·02	0·06

HGS, handgrip strength; sd, standard deviation.

a All forms of fruits include whole fruits and fruit juices.

b All forms except juices.

c Includes legumes (beans and peas), dark-green vegetables and all other vegetables.

d Legumes (beans and peas).

e Meat, poultry, eggs, seafood, nuts, seeds and soy products.

f Seafood, nuts, seeds, soy products (other than beverages) and legumes (beans and peas).

g Ratio of PUFAs and MUFAs to saturated fatty acids.

* *P* ≤ 0·05, Student's *t* test.

** *P* ≤ 0·05, analysis of covariance (ANCOVA); Adjusted for age, family number, gender, CVD medication, height and physical activity.

**Table 3.** Association between HGS and quartiles of HEI-2015

Variables, *N* (%)	HGS		*P*-value*
	PS (*N*=123)	Non-sarcopenic (*N*=78)	
HEI-2015	1st Q (*N*=50) (18·08–51·85)	37 (72·5)	13 (27·5)	0·2
	2nd Q (*N*=51) (51·86–59·98)	30 (60)	21 (40)	
	3th Q (*N*=50) (59·99–65·12)	29 (58)	21 (42)	
	4th Q (*N*=50) (65·13–88·55)	27 (54)	23 (46)	
	1st Q	37 (74)	13 (26)	0·04
	Other quartiles	86 (57)	65 (43)	
	Other quartiles	96 (63·6)	55 (36·4)	0·3
	4th Q	27 (54)	23 (46)	
	1st Q	37 (74)	13 (26)	0·06
	4th Q	27 (54)	23 (46)	

HGS, handgrip strength; Q, quartile.

* *P* < 0·05; *X^2^* test.

**Table 4.** Logistic regression: PS

Variables	Crude model	Model I*	Model II**
	OR 95 % (CI)	OR 95 % (CI)	OR 95 % (CI)
Total fruits^a^	1⋅00 (0⋅61–1⋅63)	1⋅00 (0⋅6–1⋅66)	1⋅02 (0⋅6–1⋅74)
Whole fruits^b^	1⋅57 (0⋅59–4⋅18)	1⋅33 (0⋅46–3⋅8)	1⋅4 (0⋅46–4⋅18)
Total vegetables^c^	0⋅78 (0⋅48–1⋅27)	0⋅8 (0⋅47–1⋅34)	0⋅75 (0⋅43–1⋅29)
Greens and beans^d^	0⋅95 (0⋅54–1⋅69)	1⋅02 (0⋅56–1⋅88)	0⋅97 (0⋅51–1⋅84)
Whole grains	0⋅53 (0⋅2–1⋅42)	0⋅42 (0⋅14–1⋅23)	0⋅32 (0⋅0⋅9–1⋅15)
Dairy	1⋅00 (0⋅9–1⋅1)	0⋅99 (0⋅89–1⋅1)	1⋅00 (0⋅89–1⋅12)
Total protein foods^e^	0⋅87 (0⋅64–1⋅17)	0⋅89 (0⋅64–1⋅23)	0⋅86 (0⋅6–1⋅23)
Seafood/plant proteins^f^	0⋅96 (0⋅78–1⋅19)	0⋅97 (0⋅77–1⋅22)	1⋅04 (0⋅81–1⋅34)
Fatty acids^g^	0⋅92 (0⋅84–1⋅00)	0⋅89 (0⋅81–0⋅98)	0⋅9 (0⋅81–0⋅99)
Refined grains	0⋅96 (0⋅86–1⋅07)	0⋅93 (0⋅82–1⋅04)	0⋅9 (0⋅79–1⋅03)
Sodium	1⋅00 (0⋅93–1⋅08)	1⋅01 (0⋅93–1⋅09)	0⋅99 (0⋅91–1⋅08)
Added sugars	0⋅91 (0⋅84–0⋅98)	0⋅88 (0⋅8–0⋅96)	0⋅9 (0⋅82–0⋅99)
Saturated fats	0⋅91 (0⋅83–0⋅99)	0⋅9 (0⋅82–0⋅99)	0⋅91 (0⋅83–1⋅01)
HEI-2015 model			
Quartile 1	1⋅00	1⋅00	1⋅00
Quartile 2	0⋅5 (0⋅22–1⋅17)	0⋅54 (0⋅22–1⋅33)	0⋅58 (0⋅23–1⋅47)
Quartile 3	0⋅49 (0⋅21–1⋅13)	0⋅57 (0⋅23–1⋅39)	0⋅66 (0⋅26–1⋅65)
Quartile 4	0⋅41 (0⋅18–0⋅96)	0⋅26 (0⋅1–0⋅65)	0⋅31 (0⋅12–0⋅81)
*P*-trend	0⋅05	0⋅008	0⋅03

a All forms of fruits include whole fruits and fruit juices.

b All forms except juices.

c Includes legumes (beans and peas), dark-green vegetables and all other vegetables.

d Legumes (beans and peas).

e Meat, poultry, eggs, seafood, nuts, seeds and soy products.

f Seafood, nuts, seeds, soy products (other than beverages) and legumes (beans and peas).

g Ratio of PUFAs and MUFAs to saturated fatty acids.

* Adjusted for age and gender.

** Adjusted for age, family number, gender, CVD medication, height and physical activity.

### HEI-2015, handgrip strength and confounders

Multiple linear regression models evaluating HGS and HEI scores are shown in Table 5. There was a statistically significant positive association between HEI scores and HGS in both the unadjusted (*P*=0⋅04) and adjusted (*P*=0⋅02 and=0⋅09) models.

**Table 5.** Multiple linear regression for the association of handgrip strength with HEI-2015 and component scores

Variables	Adjusted *R*^2^	Unstandardized coefficients	CI (95 %)	*P*-value*
		B (se)		
HEI-2015				
Crude model	0·014	0·5 (0·02)	(0·00–0·1)	0·04
Model I^†^	0·47	0·04 (0·02)	(0·007–0·08)	0·02
Model II^‡^	0·53	0·03 (0·02)	(−0·005–0·06)	0·09
Total fruits^a^				
Crude model	−0·005	−0·08 (0·46)	(−1·00–0·83)	0·86
Model I^†^	0·45	0·13 (0·34)	(−0·55–0·8)	0·71
Model II^‡^	0·52	0·06 (0·33)	(−0·59–0·7)	0·87
Whole fruits^b^				
Crude model	−0·0003	−0·83 (0·86)	(−2·52–0·86)	0·34
Model I^†^	0·45	0·14 (0·64)	(−1·12–1·4)	0·83
Model II^‡^	0·52	0·07 (0·61)	(−1·13–1·27)	0·9
Total vegetables^c^				
Crude model	0·001	0·46 (0·42)	(−0·36–1·28)	0·27
Model I^†^	0·46	0·24 (0·31)	(−0·37–0·85)	0·44
Model II^‡^	0·52	0·3 (0·3)	(−0·29–0·88)	0·32
Greens and beans^d^				
Crude model	0·001	0·58 (0·53)	(−0·46–1·63)	0·27
Model I^†^	0·45	−0·004 (0·4)	(−0·78–0·78)	0·99
Model II^‡^	0·52	0·09 (0·38)	(−0·66–0·85)	0·81
Whole grains				
Crude model	0·03	2·29 (0·93)	(0·46–4·13)	0·02
Model I^†^	0·46	0·52 (0·71)	(−0·89–1·92)	0·47
Model II^‡^	0·52	0·55 (0·73)	(−0·88–1·98)	0·45
Dairy				
Crude model	−0·001	−0·08 (0·1)	(−0·27–0·11)	0·39
Model I^†^	0·45	0·04 (0·07)	(−0·1–0·18)	0·6
Model II^‡^	0·52	0·04 (0·07)	(−0·1–0·17)	0·6
Total protein foods^e^				
Crude model	0·03	0·69 (0·27)	(0·15–1·23)	0·01
Model I^†^	0·46	0·15 (0·21)	(−0·26–0·57)	0·47
Model II^‡^	0·52	0·13 (0·2)	(−0·28–0·52)	0·54
Seafood/plant proteins^f^				
Crude model	−0·003	0·12 (0·2)	(−0·27–0·52)	0·54
Model I^†^	0·45	−0 ·02 (0·15)	(−0·31–−0·27)	0·88
Model II^‡^	0·52	−0·15 (0·15)	(−0·44–0·14)	0·31
Fatty acids^g^				
Crude model	0·002	0·09 (0·08)	(−0·06–0·25)	0·24
Model I^†^	0·46	0·05 (0·06)	(−0·06–0·17)	0·37
Model II^‡^	0·52	0·003 (0·06)	(−0·11–0·12)	0·96
Refined grains				
Crude model	0·004	−0·14 (0·1)	(−0·34–0·06)	0·17
Model I^†^	0·45	0·05 (0·8)	(−0·1–0·2)	0·53
Model II^‡^	0·52	0·09 (0·07)	(−0·06–0·23)	0·24
Sodium				
Crude model	−0·003	0·04 (0·07)	(−0·1–0·18)	0·57
Model I^†^	0·45	0·02 (0·05)	(−0·09–0·12)	0·76
Model II^‡^	0·52	0·02 (0·05)	(−0·08–0·12)	0·72
Added sugars				
Crude model	0·02	0·15 (0·07)	(0·01–0·3)	0·04
Model I^†^	0·49	0·18 (0·05)	(0·08–0·29)	0·001
Model II^‡^	0·52	0·13 (0·05)	(0·03–0·23)	0·009
Saturated fats				
Crude model	0·03	0·2 (0·08)	(0·05–0·35)	0·01
Model I^†^	0·46	0·09 (0·06)	(−0·03–0·2)	0·14
Model II^‡^	0·52	0·05 (0·06)	(−0·06–0·16)	0·4

a All forms of fruits include whole fruits and fruit juices.

b All forms except juices.

c Includes legumes (beans and peas), dark-green vegetables and all other vegetables.

d Legumes (beans and peas).

e Meat, poultry, eggs, seafood, nuts, seeds and soy products.

f Seafood, nuts, seeds, soy products (other than beverages) and legumes (beans and peas).

g Ratio of PUFAs and MUFAs to saturated fatty acids.

† Adjusted for age and gender.

‡ Adjusted for age, family number, gender, CVD medication, height and physical activity.

* *P* < 0·1.

## Discussion

The present results show that there is a statistically significant association between diet quality and PS. Participants with PS had lower HEI scores compared with healthy controls. Even after adjusting HGS – our surrogate parameter for PS – for several covariates, it was still significantly associated with HEI scores.

While the HEI has not yet been validated in an Iranian population, the main components of Iranian food are comparable to the US, Europe and the Middle East: refined grains like white rice and bread, potatoes and hydrogenated fats^(25)^, so HEI could be used as a good indicator of dietary intake status in the Iranian population. Based on our knowledge, this is the first study to assess the relationship between HEI and PS. In the present study, we used EWGSOP2 cutoff-values, which have been reported to be good indicators in Iranian populations^(26)^.

According to Xu *et al.*^(27)^, diet quality evaluated by HEI-2005 also had a significant association with enhanced physical performance. Consistent with our findings, Rahi *et al.*^(28)^ found that adherence to the Canadian-HEI in addition to an active lifestyle might prevent muscle strength reduction in diabetic patients. Hashemi *et al.*^(10)^ suggest that a Mediterranean dietary pattern is associated with low odds of sarcopenia in older adults. The Mediterranean diet is based on the consumption of high sources of antioxidants like MUFAs, and more fruits and vegetables that contribute to a reduction of oxidative stress, the main mechanism of sarcopenia pathogenesis^(29)^. Our findings in fruit and vegetable consumption demonstrated that PS participants consumed less than healthy older adults. The lack of statistical significance in assessing the association of fruits and vegetables with PS might be clarified by the HEI algorithm, in which consuming higher amounts of these components against what is supposed for getting maximum scores remain unconsidered.

No exact mechanisms in the relationship between HEI and PS have been described yet, but it could be mediated by physical activity as well. Most older adults adhering to a high-quality diet tend to maintain a generally healthier lifestyle and are more regularly physically active. Thus, they are less susceptible to obesity and decreased muscle strength^(17)^.

Other findings of the present study were the significant association between lower muscle strength and higher intake of added sugars and saturated fats. In aging individuals, there is an elevation of pro-inflammatory cytokines like TNF-α, IL-6 and CRP. Studies show that there is a significant association between these cytokines and low muscle mass and muscle strength^(30)^. Furthermore, NF-kB might play a role as well^(31)^. Saturated fat is one of the nutrients which stimulating inflammatory responses through the NF-kB pathway and people with a high intake of saturated fats might experience low muscle mass and weak muscle strength in comparison with those who consume high amounts of PUFAs or MUFAs as anti-inflammatory nutrients^(32)^.

Another study showed that consumption of sugar-sweetened beverages, which has increased over the last decades, causes the elevation of Homeostatic Model Assessment for Insulin Resistance (HOMA-IR), i.e., insulin resistance^(33)^. Aging is associated with insulin resistance as well. This may link with impaired muscle glucose uptake that leads to impaired intracellular energy production and, as a result, muscle weakness might occur^(34)^. Gatineau *et al.* found that feeding aging rats sucrose causes a reduction in muscle mass^(35)^. Similar to the present study, others also found that the simultaneous effect of aging and added sugar consumption may be a cause of low HGS^(36)^.

In the present study, healthy people consumed more total protein foods compared with PS subjects. Total protein foods correlated positively with muscle strength; however, this association vanished after adjustment for covariates. This lack of statistical significance might be explained in part by the imperfect HEI scoring system: as 2⋅5 ounces of total protein foods per 1000 kcal already receive the highest attainable score, further intake is going unnoticed. Various studies have already shown that people with a low intake of total protein have a lower average muscle mass and strength^(37–39)^. Decreased protein synthesis as a result of poor dietary intake might cause muscle fibre atrophy and a reduction of muscle mass leading to weak muscle strength, possibly providing the link of causality between low protein intake and low HGS^(40)^; however, there is a controversy about the association between improvement of muscle mass or weakness with dietary protein intake in many studies^(40–44)^, which warrants further research.

A strength of the present study is that participants were selected from all of Tehran's regions, thereby providing a good representation of Tehran's older adults. Furthermore, PS was detected by using the newest EWGSOP definition. Still, the present study had some limitations. First and foremost, the squeeze dynamometer used in the present study has a lower accuracy in comparison to digital ones. We used FFQ to evaluate dietary intake, and reported amounts are based on participants’ memories (which can cause both underestimation and overestimation). Finally, the present study is a cross-sectional study that cannot prove any causality, that is, it cannot determine the direct role of diet in PS.

In conclusion, giving serious consideration to adhere to dietary recommendations such as the DGAs, which we measured with the HEI-2015, might improve muscle strength in aging individuals. This finding warrants confirmation in high-quality interventional studies.
